# The pineal gland: A model for adrenergic modulation of ubiquitin ligases

**DOI:** 10.1371/journal.pone.0172441

**Published:** 2017-02-17

**Authors:** Jerry Vriend, Wenjun Liu, Russel J. Reiter

**Affiliations:** 1 Department of Human Anatomy and Cell Science, Max Rady College of Medicine, Rady Faculty of Health Sciences, University of Manitoba, Winnipeg, Manitoba, Canada; 2 Department of Pathology, Max Rady College of Medicine, Rady Faculty of Health Sciences, University of Manitoba, Winnipeg, Manitoba, Canada; 3 Department of Cellular and Structural Biology, University of Texas Health Science Center at San Antonio, Texas, United States of America; University of Brescia, ITALY

## Abstract

**Introduction:**

A recent study of the pineal gland of the rat found that the expression of more than 3000 genes showed significant day/night variations (The Hartley dataset). The investigators of this report made available a supplemental table in which they tabulated the expression of many genes that they did not discuss, including those coding for components of the ubiquitin proteasome system. Herein we identify the genes of the ubiquitin proteasome system whose expression were significantly influenced by environmental lighting in the Hartley dataset, those that were stimulated by DBcAMP in pineal glands in culture, and those that were stimulated by norepinephrine.

**Purpose:**

Using the Ubiquitin and Ubiquitin-like Conjugation Database (UUCA) we identified ubiquitin ligases and conjugases, and deubiquitinases in the Hartley dataset for the purpose of determining whether expression of genes of the ubiquitin proteasome pathway were significantly influenced by day/night variations and if these variations were regulated by autonomic innervation of the pineal gland from the superior cervical ganglia.

**Methods:**

In the Hartley experiments pineal glands groups of rats sacrificed during the day and groups sacrificed during the night were examined for gene expression. Additional groups of rats had their superior cervical ganglia removed surgically or surgically decentralized and the pineal glands likewise examined for gene expression.

**Results:**

The genes with at least a 2-fold day/night significant difference in expression included genes for 5 ubiquitin conjugating enzymes, genes for 58 ubiquitin E3 ligases and genes for 6 deubiquitinases. A 35-fold day/night difference was noted in the expression of the gene *Sik1*, which codes for a protein containing both an ubiquitin binding domain (UBD) and an ubiquitin-associated (UBA) domain. Most of the significant differences in these genes were prevented by surgical removal, or disconnection, of the superior cervical ganglia, and most were responsive, in vitro, to treatment with a cyclic AMP analog, and norepinephrine. All previously described 24-hour rhythms in the pineal require an intact sympathetic input from the superior cervical ganglia.

**Conclusions:**

The Hartley dataset thus provides evidence that the pineal gland is a highly useful model for studying adrenergically dependent mechanisms regulating variations in ubiquitin ligases, ubiquitin conjugases, and deubiquitinases, mechanisms that may be physiologically relevant not only in the pineal gland, but in all adrenergically innervated tissue.

## Introduction

The pineal gland has been extensively studied as a site in which a day/night rhythm of melatonin occurs in vertebrate species, a rhythm that depends on an intact sympathetic innervation of the gland. A recent study of Hartley et al. [1], however, provides data showing that the expression of more than 3000 genes exhibit a significant day/night variation in the pineal gland of the rat. They reported that the day/night fluctuation in many of these genes required an intact sympathetic innervation of the pineal. These investigators found that surgical removal of the superior cervical ganglia or decentralization of these ganglia prevented many of the daily variations in gene expression. We note that these investigators did not discuss the data relating to the ubiquitin proteasome system (UPS) in their dataset and did not identify components of the UPS as such; hence, we do so in the current report.

The UPS uses three enzymes sequentially to tag proteins with ubiquitin, a ubiquitin activating enzyme (E1), a ubiquitin conjugating enzyme (E2), and finally a ubiquitin ligase (E3). One established function of ubiquitination is to mark proteins for degradation by the proteasome. Ubiquitin is recycled via deubiquitinase (DUB) enzymes. Proteasomal degradation is particularly important for those proteins with a relatively short half-life. Since the Hartley report provided a dataset that contains gene expression data for E1, E2, E3 and DUBs we could examine this dataset for day/night variations in these components of the ubiquitin proteasome pathway. Diurnal variations in ubiquitin ligases and deubiquitinases associated with the enzymes required for melatonin synthesis would be expected. Since a significant night-time increase in type 2 deiodinase (Dio2) has also been found (see below) it was of interest to examine whether the ubiquitin ligases and deubiquitinases associated with Dio2 were significantly influenced by day/night changes in photoperiod as well. It was remarkable, however, to find that the Hartley dataset provided evidence that expression of many genes for ubiquitin ligases in the pineal gland, as well as those for some ubiquitin conjugases, were subject to day/night variation, and that this variation was associated with sympathetic innervation of the pineal.

## Methods

According to the description of the experimental design in Hartley et al. [1], four groups of Sprague Dawley rats (N = 18–20) were assigned to one of four groups at 5 days of age: 1. controls with no surgical procedure; 2. sham-operated controls; 3. a group in which the superior cervical ganglia were surgically removed; 4. a group undergoing bilateral decentralization of the superior cervical ganglia. The animals were maintained on a 14L/10D photoperiod cycle, where ZT0 (Zeitgeber time 0) is the time of lights on. The rats were maintained until they were 40 days of age. At this time one half of each of the four groups of animals was sacrificed at mid-day (ZT7) and one half of each group sacrificed at midnight (ZT19) for collection of pineal glands. The pineal glands of rats sacrificed at midnight would be expected to have higher N-acetyl transferase activity and greater melatonin secretion than those sacrificed at mid-day. RNA was extracted from pineal glands and RNA sequencing was used to analyze day/night differential gene expression in the four experimental groups.

Using the Ubiquitin and Ubiquitin-like database (http://uucd.biocuckoo.org) we searched the Excel files of the Hartley dataset to identify genes for ubiquitin ligases, deubiquitinases and ubiquitin conjugating enzymes. Of the genes so selected by the UUCD database we further selected those with a 2-fold difference in expression associated with the experimental variables, and a significant (p < .05) false discovery rate (FDR). The UUCD database also identified genes having ubiquitin-binding domains, ubiquitin-like domains, and ubiquitin-associated domains.

Using these statistical criteria we identified genes for 58 ubiquitin ligases, 6 deubiquitinases, 5 ubiquitin conjugating enzymes and one ubiquitin activating enzyme, whose expression was significantly increased or decreased by at least 2-fold at night. These data provide a conservative estimate of the proteins of the ubiquitin proteasome system in the pineal gland influenced by 24-hour variations in environmental lighting. We found that reducing the fold criterion to 1.5 increased the number of ubiquitin ligases whose expression was significantly influenced by the day/night variable to more than 150. We have listed the E3 ubiquitin ligases that were shown to have a 2-fold significant rise at night in Table 1. Those with a 2-fold significant decrease are shown in Table 2. Ubiquitin conjugating enzymes whose expression was significantly modified by the day/night variable are shown in Table 3. Most of the significant differences in these genes were prevented by surgical removal, or disconnection, of the superior cervical ganglia, and most were responsive, in vitro, to treatment with a cyclic AMP analog, and norepinephrine. All previously described 24-hour rhythms in the pineal require an intact sympathetic input from the superior cervical ganglia.

**Table 1:** Ubiquitin Ligases of the rat pineal gland whose expression is increased at least 2-fold at night in the Hartley dataset.

Gene symbol	Ubiquitin ligase Family	Best Fold change (Night/day)	FALSE DISCOVERY RATE	DBcAMP fold increase*	NE fold increase*
CHD5	Ring	10.13	2.47E-30	9.18	8.34
KCTD3	BTB	8.07	1.35E-112	1.72	2.49
KCNV2	BTB	6.86	3.99E-55	1.71	
LONRF1	Ring	6.62	1.14E-52	11.52	8.11
EML5	Ring DWD	6.22	5.61E-108	2.89	2.62
RLIM	Ring	4.87	7.53E-58	4.39	3.91
PDZRN3	RING	4.49	8.15E-21	2.94	2.37
BTBD9	BTB	3.89	9.38E-21	1.34	1.44
RHOBTB3	BTB	3.87	4.64E-42	3.08	4.13
KLHL30	BTB	3.81	6.94E-10	1.61	1.88
FBXO33	F Box	3.38	1.04E-39	2.02	1.66
KLHL14	BTB	3.26	2.791E-05		
LNX1	Ring	3.16	4.19E-15	1.46	1.52
Wipi2	Ring	2.96	3.34E-33	1.91	2.11
UBR4	UBR Box	2.81	2.07E-152	2.21	1.88
KCTD8	BTB	2.77	1.46E-04	1.99	
WDR63	Ring DWD	2.77	2.13E-06		
HERC4	Hect	2.76	6.01E-18	4.37	3.56
KLHL29	BTB	2.71	6.39E-20	1.76	2.01
BCL6	BTB	2.53	3.93E-12		
FBXL17	F Box	2.51	9.46E-17	1.21	1.19
RNF122	Ring	2.45	1.37E-11	1.72	1.63
IRF2BPL	Other	2.33	5.02E-07	1.80	1.56
ASB1	Socs	2.32	1.33E-09	1.55	1.46
CABP1	Ring	2.30	1.58E-16	2.57	2.88
HERC6	Hect	2.29	2.38E-02	1.54	1.23
OSTM1	Ring	2.26	1.16E-35	1.63	1.21
SPSB1	Socs	2.22	7.52E-03	1.67	1.36
SPSB2	Socs	2.18	6.09E-16	2.10	2.01
FBXO6	F Box	2.16	6.89E-24		
WDR90	Ring DWD	2.15	1.94E-19		
KLHL4	BTB	2.11	8.53E-09		
ZBTB47	BTB	2.10	5.21E-17	1.36	1.53
TRIM26	Ring	2.04	1.76E-17	1.58	1.42
ZBTB33	BTB	2.04	7.55E-17	1.73	1.51
RHOBTB2	BTB	2.01	6.16E-18	2.32	2.46
SPSB4	Socs	2.00	7.10E-04	1.63	1.54

*with significant FDR

**Table 2:** Ubiquitin ligases of the rat pineal gland whose expression is decreased at least 2-fold at night in the Hartley dataset.

Gene symbol	Ubiquitin ligase family	Best Fold Change (Night/day)	False Discovery Rate	DBcAMP fold change	NE fold change
Wdr89	Ring/DWD	-5.06	1.26E-62	-2.08	-1.85
KCNA2	BTB	-3.95	2.07E-19		
TRIM9	Ring	-3.91	6.10E-60		
Kbtbd12	BTB	-3.77	8.84E-04		
Tle1	Ring/DWD	-3.62	1.54E-51	-1.95	-2.33
DTX4	Ring	-3.39	2.30E-19		
ZFP238	BTB	-3.31	8.54E-30		
Abtb2	BTB	-2.95	6.91E-14		
KCTD19	BTB	-2.73	1.13E-03		
TRIM45	Ring	-2.71	1.82E-11		
RNF19b	Ring	-2.60	2.27E-33		
KLHL10	BTB	-2.40	1.64E-02		
Kcng4	BTB	-2.32	9.37E-06	-1.87	-2.73
KLHL33	BTB	-2.32	2.94E-03	-2.51	-1.81
PELI2	Ring	-2.29	9.69E-04		
Daw1	Ring/DWD	-2.28	3.14E-04		
Traf3ip2	Ring/U-box	-2.28	1.77E-08		
FBXO23	F Box	-2.25	9.29E-04		
TRAIP	Ring	-2.23	6.74E-04	-2.03	-1.52
Bard1	Ring	-2.19	7.34E-08		
MARCH4	Ring	-2.15	2.66E-04	-3.45	-2.89
KLHL36	BTB	-2.14	3.83E-05		
RNF182	Ring	-2.13	8.53E-03		

**Table 3:** Ubiquitin E2 conjugases of the rat pineal gland whose expression is increased or decreased at night.

Gene symbol	Fold Change (Night/day)	FDR (Night/day)	Fold Change (DBcAMP)	FDR DBcAMP
Ube2o	**3.05**	**6.16E-16**	3.32	2.10E-103
Ube2ql1	**2.84**	**9.23E-03**	NA	Ns
Ube2f	**2.45**	**2.76E-32**	1.77	9.48E-26
Ube2q2l	**2.25**	**3.57E-06**	NA	Ns
Ube2t	**-2.22**	**1.86E-02**	NA	Ns

Furthermore, we identified the ubiquitin ligases, ubiquitin conjugases, and deubiquitinases significantly modified by DBcAMP (dibutyryl-cAMP) or norepinephrine (NE) *in vitro* as reported in the Hartley dataset. Tables 4 and 5 list the ubiquitin ligases significantly increased or decreased by addition of DBcAMP or NE to pineal glands *in vitro*. We also discuss the deubiquitinases that were identified as significantly modified by the light/dark cycle and DBcAMP or NE.

**Table 4:** Ubiquitin ligases of the rat pineal gland whose expression is increased by dbcAMP* or NE *in vitro*.

Gene symbol	Fold Change dBcAMP	False Discovery Rate	Fold change NE	False Discovery Rate
LONRF1	11.52	6.65E-174	8.11	7.6E-142
CHD5	9.18	1.62E-30	8.34	2.79E-28
HERC4	4.37	4.38E-270	3.56	5.22E-66
ZBTB16	3.53	8.6E-07	1.64	ns
RHOBTB3	3.08	1.75E-63	4.13	1.2E-149
KCNC1	3.04	8.18E-15	1.88	1.07E-05
PDZRN3 (LNX3)	2.94	1.35E-16	2.37	8.98E-19
EML5	2.90	1.01E-120	2.63	6.23E-51
RNF43	2.65	4.84E-09	2.21	4.41E-10
TRIM2	2.61	2.05E-96	2.29	6.3E-88
LNX2	2.50	7.75E-07	1.75	1.50E-04
CISH	2.46	8.25E-06	2.33	5.95E-12
RHOBTB2	2.32	1.45E-22	2.46	1.78E-26
BIRC2	2.27	9.71E-31	1.75	2.34E-19
UBR4	2.21	2.50E-59	1.88	9.88E-89
FBXO2	2.18	9.05E-04	1.62	1.70E-02
SPSB2	2.10	6.91E-12	2.01	2.64E-14
RC3H1	2.08	1.89E-20	1.62	1.47E-15
FBXO33	2.02	4.94E-65	1.67	6.99E-12
PCGF3	2.02	2.90E-23	1.65	1.89E-12
NEURL1	2.02	1.75E-03	1.88	5.01E-04
SALL1	2.01	1.24E-22	2.17	3.32E-38
ZMIZ1	2.00	1.43E-32	1.88	3.81E-34
AREL1	2.00	6.12E-39	1.86	4.03E-21

*this table includes all ligases increased at least 2-fold by DBcAMP in the Hartley dataset

**Table 5:** Ubiquitin ligases of the rat pineal gland whose expression is decreased by dbcAMP* or NE *in vitro*.

Gene symbol	Fold Change cAMP	False Discovery Rate	Fold change NE	False Discovery Rate
MARCH4	-3.45	2.49E-11	-2.88	1.57E-10
KCNB2	-3.12	8.98E-19	-3.14	1.05E-18
RNF222	-2.63	1.19E-03	-1.63	ns
KLHL33	-2.50	1.54E-11	-1.81	1.82E-06
KCNS1	-2.22	1.48E-02	-1.10	ns
KCNA4	-2.17	1.63E-02	-1.10	ns
PDZD4	-2.08	9.78E-16	-1.91	1.50E-23
WDR89	-2.08	2.66E-17	-1.85	9.10E-14
BRCA2	-2.08	5.23E-14	-1.67	1.24E-09
TRAIP	-2.04	1.02E-02	-1.52	ns
ZBTB45	-2.04	4.66E-07	-1.28	ns
BTBD17	-2.04	3.11E-02	-1.35	ns

*this table includes all ligases increased at least 2-fold by DBcAMP in the Hartley dataset

We examine the Hartley dataset for evidence that the pineal gland is a useful model for studying adrenergically-dependent mechanisms regulating variations in ubiquitin ligases, ubiquitin conjugases, and deubiquitinases, mechanisms that may be physiologically relevant in all adrenergically-innervated tissue. Furthermore, based on current literature, we reinterpret the Hartley dataset in terms of the role that the proteasome plays in bringing about the daily adrenergically-induced day/night changes in proteins of the ubiquitin proteasome pathway.

## Results and discussion

In the Hartley dataset, genes whose expression in the pineal gland varied on a day/night basis include ubiquitin ligases that control melatonin synthesis, that regulate type 2 deiodinase, that modulate the Sonic Hedgehog (SHH) pathway, that regulate potassium channels, and many E3 ligases whose substrates are not documented. Also included among the genes in the pineal gland whose expression varied greatly on a day/night basis was the gene *SIK1* (salt-inducible kinase 1), which codes for a protein that contains both a ubiquitin-binding domain and a ubiquitin-associated domain and may modulate the interaction of ubiquitinated substrates with the proteasome.

### Adrenergic and proteasome regulation of melatonin synthesis

**Fig 1:**
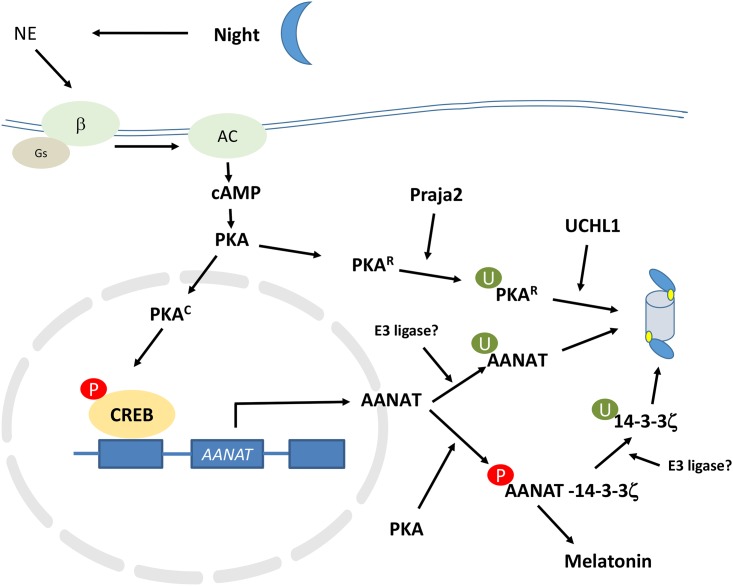
B-adrenergic innervation and ubiquitin proteasome system regulation of AANAT and melatonin concentrations in the pineal gland. R—regulatory component of PKA; C—catalytic subunits of PKA; β - Beta adrenergic receptor; Ac—Adenyl cyclase; Gs—G stimulatory protein that activates adenyl cyclase; Praja2 –ubiquitin ligase coded for by *PJA2* gene; UCHL1—Ubiquitin C-terminal hydroxylase, a deubiquitinase enzyme; CREB—cyclic AMP response element binding protein; AANAT—aralkylamine N-acetyltransferase; U—ubiquitin; P–phosphate; 14-3-3ζ –a protein kinase inhibitor protein coded for by the *YWHAZ* gene

The role of adrenergic innervation and the cAMP/PKA pathway in stimulating AANAT (serotonin N-acetyltransferase) transcription has been well described by the Klein’s research group [2–4]. They have also provided evidence of the involvement of the ubiquitin proteasome system in regulation of the rate limiting enzyme for melatonin synthesis, AANAT; they concluded that cAMP regulates AANAT through stimulation of transcription, on one hand, and inhibition of proteasomal degradation on the other [5]. Huang et al. [6] found that proteasome inhibition resulted in a substantial accumulation of AANAT in the pineal. While this study provided evidence that proteasomal degradation contributes to the regulation of this enzyme, it did not identify the ligases and deubiquitinases that regulate AANAT degradation. The report also did not identify the mechanism by which light suppresses melatonin levels in the pineal. The ubiquitin ligases and deubiquitinases associated with the synthetic enzymes for melatonin production thus remain unknown but could be among those identified as statistically influenced by the day/night variables in the Hartley dataset. Since gene expression of AANAT in the Hartley study was increased more than one hundred and seventy fold in rats sacrificed at night (see their supplemental table S1), their dataset was useful for studying concurrent changes in components of the UPS. In Fig 1 we show the dual role of cAMP in AANAT regulation and illustrate the potential functions of ubiquitin ligases and the deubiquitinase Uchl1 in stabilization/destabilization of AANAT protein.

One protein that binds to AANAT and protects it from degradation is the protein 14-3-3ζ (coded for by the gene *Ywhaz*) [7, 8]. This binding requires prior phosphorylation of 14-3-3ζ. While the ubiquitin ligase for at least one other member of the 14-3-3 family has been identified as Cop1 [9, 10], the ubiquitin ligase for 14-3-3ζ has apparently not been identified. It should be noted that the Hartley dataset shows that expression of *Ywhaz* was significantly increased at night (1.70 fold, p < 9.05^E-09^). This rise was prevented by surgical removal of the superior cervical ganglia or by their decentralization (Hartley supplemental dataset S1). *Ywhaz* expression was significantly stimulated in culture by DBcAMP and by NE. These data support the view that 14-3-3ζ may be part of an adrenergically-dependent mechanism regulating AANAT stability [7]. Other members of the 14-3-3 family, 14-3-3 epsilon, 14-3-3 beta, 14-3-3 gamma, 14-3-3 tau and 14-3-3 sigma showed no significant day/night difference in the Hartley dataset.

14-3-3ζ interacts with many other proteins. These include specific interactions with at least 300 proteins, including subunits of the proteasome. Although these data are hidden away in supplementary data tables some of them are quite remarkable. According to the data of Ge et al. [11] 14-3-3ζ interacts with six subunit proteins of the beta ring of the proteasome and with ten protein subunits of the lid of the proteasome.

Klein et al. [8] have suggested a model in which 14-3-3 proteins interact with phosducin as part of the photosensitivity mechanism. In the Hartley data set, expression of the phosducin gene (*Pdc*) shows a 2-fold increase during the day at a very high level of significance, thus supporting the Klein model. Phosducin has been reported to interact with a subunit of the proteasome, Sug1 (aka Trip1, Psmc5) [12].

In the Klein model, adrenergic stimulation of cAMP/PKA results in the phosphorylation of AANAT and its protection by 14-3-3ζ [7]. Since PKA (protein kinase A) is subject to regulation through degradation by ubiquitin ligases and by deubiquitinases, variations in the activity of ligases and DUBs may also influence AANAT levels. The ubiquitin ligase Praja2 (Pja2) has been reported to regulate PKA stability by controlling degradation of the regulatory (R) component of PKA [13]. The deubiquitinase Uchl1 (ubiquitin C-terminal hydrolase L1) also contributes to PKA stability [14]. In the Hartley dataset nighttime expression of *Uchl1* was significantly decreased by superior cervical ganglionectomy. It appears that the turnover of the R component of PKA is regulated by a balance of ubiquitination and deubiquitination. More extensive data on the role of Pja2 and Uchl1 in AANAT activity would be useful. While Pja2 also interacts with the E2 conjugase, *UBE2D2* (aka UBCH5B) [15], expression of the genes for *Pja2* and *UBE2D2*, although significantly increased at night in the Hartley dataset, were not elevated by 2-fold.

In Fig 1, we summarize the interaction of 14-3-3ζ with AANAT and the proteasome. This figure integrates the Klein model with data showing the relationship of 14-3-3ζ with the proteasome. It also illustrates a role for the ubiquitin ligase Praja2 in AANAT turnover by controlling the regulatory unit of PKA [13].

### Adrenergic and proteasomal regulation of the melatonin receptor complex

*Ostm1* (osteopetrosis associated transmembrane protein 1) (aka *GIPN*) is a gene that codes for an ubiquitin ligase involved in the degradation of G proteins via the proteasome [16]. Among these is the protein RGS20 (regulator of G-protein signaling 20) [16, 17]. RGS20 is part of the MT1 melatonin receptor GPCR complex [18]. The expression of *Ostm1* was enhanced 2-fold at nighttime in the Hartley dataset (Table 1). It was also significantly enhanced (by 1.62-fold) by DBcAMP. Thus, the Hartley dataset provides support for the concept of adrenergic and photoperiodic regulation of the melatonin receptor. Alonso and Friedman [19] have reviewed and illustrated a general mechanism showing the role of the UPS in G protein-coupled receptor recycling.

### Adrenergic regulation of *SIK1* expression

Another protein reported to play a role in adrenergic-regulation of AANAT in the rat pineal gland is the salt-inducible kinase, SIK1. Bailey et al. previously noted that expression of the *Sik1* gene was stimulated at night in rat pineal glands [20]. Kanyo et al. [21] subsequently reported a nocturnal induction of SIK1 in cultured pinealocytes. These investigators also found that treatment with norepinephrine or DBcAMP also stimulated *Sik1* transcription in pineal cell culture. Furthermore they found that overexpressing SIK1 had an inhibitory effect on AANAT induction, as well as on expression of other cAMP regulated genes.

In the Hartley dataset *Sik1* gene expression was elevated approximately 35-fold at night (FDR < 2.96 E^-192^), but not in the pineals of rats lacking superior cervical ganglia, nor in those with decentralized superior cervical ganglia (see their supplemental dataset S1). *Sik1* gene expression in pineals of sham-operated rats was again increased greater than 35-fold. In this dataset *Sik1* was stimulated by DBcAMP or by NE by approximately 12-fold (see their supplemental dataset S3). Thus the Hartley data on *SIK1* gene expression confirm the earlier findings of Kanyo et al. [21] on SIK1 protein concentrations in rat pinealocytes in culture. The large night-time increase in *Sik1* expression and the very high level of statistical significance in the Hartley dataset suggest a significant physiological role for SIK1 in the pineal gland. *SIK1*, however, was not discussed by Hartley et al. [1] even though their Table 1 lists it as among 12 genes whose change in expression was greater than 32-fold due to day/night variation. This table also shows that this gene exhibits relatively high night-time expression in the pineal gland compared to expression in control mixed tissues.

SIK1 belongs to a family of proteins that contain a UBD domain and a UBA domain. These proteins bind to polyubiquitinated proteins through the UBA domain [22–24]. In the model of Su and Lau [23] proteins with both UBA and UBL domains act as adaptors, or shuttles, which link ubiquitinated proteins to the proteasome (see their Fig 2) and facilitate their degradation. The functional role of the UBD and UBA domains have not been well characterized for SIK1. The interaction of SIK1, through its UBA domain, with other polyubiquitinated proteins suggests a regulatory role in proteasomal degradation of proteins.

**Fig 2:**
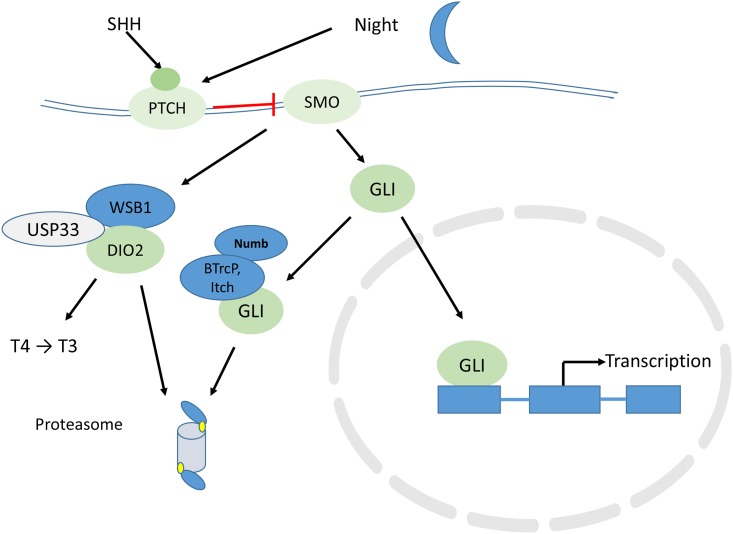
Patch and ubiquitin proteasome system regulation of Dio2 in the pineal gland. SHH—Sonic Hedgehog Homolog protein; PTCH—patched homolog protein, a receptor for SHH; SMO—smoothened protein, a receptor that interacts with the PTCH receptor; GLI—glioma-associated oncogene protein, a transcription factor; WSB1 –WD repeat and SOCS box-containing protein, a ubiquitin ligase; DIO2 –type two deiodinase; USP33—ubiquitin specific peptidase 33, a deubiquitinase.

In the pineal gland SIK1 may contribute to the turnover of proteins whose concentrations vary, on a day/night basis under the influence of beta- adrenergic innervation. A mechanism not previously considered is that SIK1 facilitates protein turnover in the pineal by modifying their interaction with the proteasome through its UBD and UBA domains. It remains to be determined whether the SIK1 protein is secreted into the blood vascular system or into the CSF (cerebrospinal fluid) to have an impact on the degradation of proteins outside the pineal gland. According to the report of Kanyo et al. [21] another gene regulated by SIK1 is the gene for type two deiodinase (*Dio2*).

### Adrenergic and ubiquitin ligase control of type two deiodinase (Dio2) in the rat pineal gland

In the Hartley dataset type two deiodinase gene (*Dio2*) expression was increased at least 14-fold (p < 4.56 ^E-23^) at night (Hartley supplemental dataset S1). Evidence that Dio2 is increased at night in the pineal gland of rats has previously been reported [25, 26]. The day/night rhythm in Dio2 is thought to provide a mechanism for regulating tissue levels of T3. Klein et al. [27] point out that T3 may have an impact on the sensitivity of pineal cells to adrenergic stimulation. As previously shown [25], the Hartley dataset confirms that the nighttime increase in *Dio2* is prevented by surgical removal or decentralization of the superior cervical ganglia, indicating that the large rise in *Dio2* at night is dependent on an intact adrenergic innervation [25, 28]. Furthermore the *in vitro* findings in the Hartley dataset (supplemental dataset S3) provide further support for an adrenergic/cAMP mediated increase in Dio2. Both DBcAMP and NE treatment elevated *Dio2* gene expression in the rat pineal [29, 30].

Proteasome regulation of Dio2 has been documented. Two ubiquitin ligases for this enzyme have been identified in a variety of tissues [31, 32], WSB1 and TEB4 (aka MARCH6, aka RNF176), as well as one deubiquitinase. According to Sagar et al. [33] the ubiquitin ligase WSB1 (WD repeat and socs box-containing 1) and the deubiquitinase USP33 (aka VDU-1, the von Hippel-Lindau protein-interacting deubiquitinase-1) [34] are bound to the Dio2 protein simultaneously, providing a switch mechanism that rapidly regulates stability of the protein. The ubiquitin conjugase UBE2G2 (aka UBC-7) interacts with WSB-1 to ubiquitinate Dio2 [33]; the same conjugase interacts with TEB4 [31]. The expression of *Wsb1* and *Usp33* genes in the pineal gland of the Hartley summary were recorded in their supplemental dataset S1 as significantly increased at night. The expression of *Ube2g2* gene was also significantly elevated at night in the Hartley dataset, but not at the 2-fold level.

Expression of the ubiquitin ligase TEB4 was also significantly elevated at night (at a high level of significance, FDR < 1.97^E-16^). Thus, the Hartley dataset support the Sagar model in which ubiquitin ligases and deubiquitinases regulate the stability of the Dio2 protein. It appears, therefore, that the stability of the Dio2 protein, as well as the transcription of the *Dio2* gene, are strongly regulated by an adrenergic mechanism in the rat pineal. Since melatonin administration has been shown to inhibit expression of the *Dio2* gene in the hypothalamus of mammals [35, 36], it would be interesting to determine whether melatonin inhibits expression of the *Dio2* gene within the pineal gland.

In other tissues it has been noted that the Sonic Hedgehog pathway (SHH) increases ubiquitination of Dio2 via induction of the E3 ligase WSB1 [33, 37, 38]. Thus the SHH pathway modulates thyroid hormone activation. We would therefore expect some evidence for day/night variation in the SHH pathway in the Hartley dataset. By examining their report, we do find that expression of the SHH receptor, Ptch1 (patched 1), is increased at night, by about 18-fold (FDR < 5.89^E-141^). A transcription factor in the SHH pathway, GLI2 was also reported in the Hartley dataset as increased at night (2.5 fold, FDR < 1.06^E-28^). Fig 2 illustrates the signaling cascade put in motion by SHH activation of its receptor, Ptch1, and shows the relationship of Ptch1 stimulation to the activity of Dio2. The nighttime increase in Ptch1 was not observed in animals whose superior cervical ganglia were surgically removed. An important (although heretofore unmentioned) contribution of the Hartley dataset is the evidence showing adrenergic regulation of the Ptch1 receptor. The *in vitro* results show an approximately 15-fold increase in *Ptch1* expression following stimulation with either DBcAMP or NE.

### The SIK1 model

**Fig 3:**
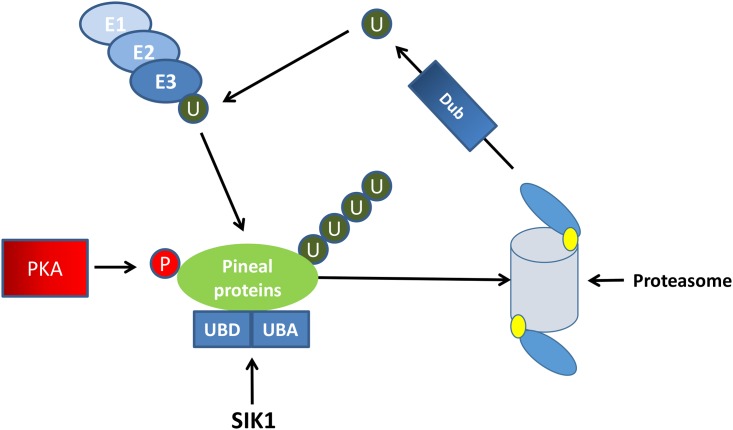
A model of SIK1 function in the pineal gland. SIK1 –salt-inducible kinase 1; E1 –ubiquitin activating enzyme; E2 –ubiquitin conjugase; E3 –ubiquitin ligase; U—ubiquitin; PKA–protein kinase A; P–phosphate; DUB–deubiquitinase; UDB–ubiquitin binding domain; UBA–ubiquitin associated domain.

Fig 3 illustrates how the SIK1 protein, via its UBD and UBA domains, could modulate the degradation of proteins, including AANAT and Dio2, by the proteasome. This model provides for a mechanism by which SIK1 facilitates the daily degradation of proteins that vary according to a circadian rhythm. A role for SIK1 in entraining circadian rhythms by inhibiting cAMP induced changes in the clock gene, *Per* (period), in the hypothalamus has been presented by Jagannath et al. [39]. Their data suggest a negative feedback effect of SIK1 on cAMP induced transcription at the cAMP response element. The Hartley statistics showing at least a 35-fold increase at night, an increase prevented by removal of the superior cervical ganglia, suggest this protein could also have a key role in the physiology of the pineal gland as a protein linking ubiquitinated proteins to the proteasome.

### Unexplained role of thyroid peroxidase in the pineal gland

As an aside, we also note the expression of the thyroid peroxidase gene (*TPO*), particularly at night, in the Hartley dataset. This enzyme oxidizes iodide to the iodine radical immediately prior to reaction with tyrosine on thyroglobulin in thyroid follicles. Its role in tissues other than the thyroid gland is unknown. Thus, the expression of *TPO* and its 4-fold day/night variation in these data is unexplained. Further examination of the dataset shows that the expression of the gene for thyroglobulin (*TG*) was also significantly increased at night (P < .0001). The higher nighttime expressions of *TPO* and *TG*, furthermore, were shown to be dependent on intact superior cervical ganglia.

While there is no evidence that thyroid hormones are synthesized in the pineal, the expression of *TPO* and *TG* genes at night indicate this may occur. Thyroid hormones, T3 and T4, however, have been measured in the rat pineal gland [40]. The requirements of extrathyroid synthesis of thyroid hormones have previously been discussed [41] and include, in addition to TPO and TG, a sodium iodide symporter (NIS) and the dual oxidases, Duox1 and Duox2, which contribute to hydrogen peroxide production necessary for thyroid hormone synthesis. While this does not establish that thyroid hormone synthesis occurs in the pineal gland, it does suggest that some of the basic requirements for thyroid hormone synthesis are found in this gland.

### Adrenergic regulation of ubiquitin conjugases in the pineal gland

The expression of genes for several ubiquitin conjugating enzymes were reported as significantly increased at night at the 2-fold level in the Hartley dataset, at a high level of statistical significance (see Table 3). These include *Ube2o* (3.04-fold), *Ube2ql1* (2.84-fold), *Ube2f* (2.45-fold), and *Ube2q2l* (2.25-fold). *Ube2s* was significantly elevated by 1.85-fold at night. A nighttime rise in *Ube2s* was previously reported [20]. A significant depression of 2.22-fold was noted in the gene for the ubiquitin conjugase *Ube2*t. None of these nighttime changes were significant in rats subjected to superior cervical ganglionectomy or to decentralization of the superior cervical ganglia. *Ube2o*, *Ube2f* and *Ube2s* were significantly stimulated *in vitro* by either DBcAMP or NE. Thus the Hartley dataset provides strong evidence for adrenergically-mediated increases of expression of these ubiquitin E2 conjugases in the rat pineal gland. While we consider this a major finding, it was not discussed by Hartley et al. [1]. Of genes for the ubiquitin conjugases indicated above, the one most significantly increased by cAMP/adrenergic stimulation is *Ube2o*. It was elevated 3.3-fold by DBcAMP (FDR = 2.10^E-103^) and 2.86-fold by NE (FDR = 2.19^E-76^). UBE2O is described as a hybrid ubiquitin ligase with both E2 conjugase and E3 ligase activity [42]. Since there are more than 30 ubiquitin conjugases and as many as 1000 ubiquitin ligases [43] the ratio of ligases to conjugases can be estimated to be in the range of 20:1 to 25:1. Thus it is likely that in addition to transferring ubiquitin to various E3 ligases, UBE2O also has a number of unique substrates that it can ubiquitinate directly. Therefore its impact on downstream signaling would be expected to be greater than that of other ubiquitin conjugases. Although little is known concerning the substrates of UBE2O, its hybrid character gives it the potential to modulate a variety of cellular processes within the pineal gland. Zhang et al. [42] reported that the signal transduction protein SMAD6 is a substrate of UBE2O. UBE2O also interferes with TRAF6 (TNF receptor-associated factor)-mediated NF-κB signaling [44]. TRAF6, itself an E3 ubiquitin ligase, modulates various aspects of the immune response [45].

The E2 conjugase, UBE2F (see Table 3), also has the potential to influence the activity of various E3 ligases and hence the degradation of various proteins in the pineal gland. UBE2F is one of the conjugases required to activate cullin-ring E3 ligases by attaching NEDD8 to them [43, 46].

It should be noted that the Hartley dataset provides some evidence for day/night differences in expression of the gene for one of the E1 ubiquitin activating enzymes recognized by Van Wijk and Timmers [43]. The expression of *Uba2* was depressed 2.47 fold at night at a high level of significance (FDR < 1.03^E-48^). Its expression was also significantly depressed by DBcAMP and NE, but not at the 2-fold level. The expression of *Uba6*, another E1 activating enzyme, was also significantly reduced by dBcAMP at a modest level.

Since many ubiquitin E3 ligases are also modulated by adrenergic cAMP signaling in the Hartley dataset (see below), they show that this pathway regulates the activity of both E2 and E3 enzymes (and possibly E1 enzymes) of the ubiquitin proteasome pathway in the pineal gland. The interaction of E2 and E3 enzymes during adrenergic stimulation in the pineal gland has yet to be determined. Furthermore, the Hartley dataset shows that the rat pineal gland is a novel model for studying adrenergic regulation of ubiquitin conjugases and adrenergic regulation of ubiquitin ligases and their substrates (see below). Adrenergic mechanisms regulating E2 conjugases probably have a cascading effect since E2 conjugases transfer ubiquitin to several E3 ligases. Much is to be learned of the function of signaling networks in the pineal gland whose proteins are modulated by E2 and E3 enzymes.

### Ubiquitin ligases as adrenergic targets in the pineal gland

The Hartley dataset identifies numerous genes that are stimulated by DBcAMP and/or NE. One of these genes is *ADRB1*, the gene for the beta-1 adrenergic receptor. In pineal organ culture both DBcAMP and NE increased the expression of this gene more than 2-fold. Below we discuss the stimulation or inhibition of various ubiquitin ligases by DBcAMP and/or NE as reported in the Hartley report, keeping in mind that beta-adrenergic receptors themselves are subject to recycling by a mechanism involving B-arrestin [47] and ubiquitin ligases [19, 48].

#### The LNX ubiquitin ligases

We note from the list of E3 ubiquitin ligases in the Hartley dataset that two members of the LNX (ligand of numb protein X) family of ubiquitin ligases [49] were significantly elevated at night (Table 1), while another one (*Lnx4*) was increased during the day. *Lnx1* expression was stimulated 3-fold and *Lnx3* expression rose 4.5 fold at night, while *Lnx2* was not significantly influenced by the light:dark cycle. *Lnx1*, *Lnx2* and *Lnx3* expression were all significantly stimulated by DBcAMP and NE in vitro in the Hartley study (see their supplemental Table S3). *Lnx2* and *Lnx3* were increased by DBcAMP and by NE by more than 2-fold, at a high level of significance (Table 4). Thus, the findings provide strong evidence for noradrenergic stimulation of one or more LNX ubiquitin ligases during the night.

One of the substrates of LNX1 is the protein NUMB [50]. Its role in the SHH pathway was reviewed recently [51] and is shown in our Fig 2. NUMB, together with the ubiquitin ligase, ITCH (itchy E3 ubiquitin protein ligase), promotes the ubiquitination and degradation of GLI (GLI1), opposing the action of SHH. By regulating GLI, NUMB has the potential of regulating rhythms of cell proliferation. Numb also inhibits MDM2 (mouse double minute 2) activity and thereby increases the activity of p53 [51]. By contributing to the regulation of p53, NUMB has the potential of influencing rhythms in growth, DNA repair, apoptosis, inflammation and autophagy (illustrated in Flores et al. [51]. In addition, NUMB acts as an inhibitor of NOTCH signaling [51].

The E3 ligase LNX3 (aka SEMCAP3, aka PDZRN3) is associated with the synapse where it modulates the expression of a receptor tyrosine kinase (MuSK) specific to neuromuscular junctions [52]. There is also evidence that LNX3 binds to the glutamatergic receptor subunit GluR2 [53]. It also interacts with the E2 conjugase UBCH5B (aka UBE2D2) [52].

#### The KCTD family of ubiquitin ligases and potassium channels

Also influenced by the day/night light dark cycle in the Hartley dataset were 3 members of the KCTD family, which are reported to be substrate-specific adaptors for Cullin 3 ubiquitin ligases [54]. The KCTD family, a group of proteins with a potassium channel tetramerization domain, has various functions including acting as ubiquitin ligases [55]. *Kctd3* and *Kctd8* were significantly elevated at night by 8.07-fold and 2.77-fold respectively, in the Hartley dataset (Table 1) at a very high level of significance. Both were significantly stimulated by DBcAMP or NE (Hartley Supplemental Table S3). The substrates of all the KCTD ligases have not been well characterized. KCTD3 is reported to bind to the protein HCN-3 (hyperpolarization-activated cyclic nucleotide-gated channel complex) [56] and to regulate its expression on the cell surface.

KCTD11, however, is a member of the KCTD family that has been reasonably well characterized [57]. *Kctd11*, which was not significantly influenced by the light/dark cycle in the Hartley dataset is an E3 ligase which influences the activity of the GLI transcription factor by regulating the degradation of HDAC1, a deacetylase enzyme [58]. *Kctd19* significantly rose during the day (fold change = 2.73).

A recent report from the research group of Cienchanover listed several potassium channel components as ubiquitin E3 ligases (see their supplemental table S4) [59]. In Table 1, we see that the expression of one of these components, Kcnv2, was increased 6.86-fold at night, at a high level of significance (FDR <3.99^E-55^). Thus the Hartley dataset provides strong evidence that day/night differences in potassium channel components and the cAMP/adrenergic modulation of these channels in the pineal gland depend on the variable activity of E3 ubiquitin ligases associated with these ion channel components.

#### Other ubiquitin ligases increased at least 2-fold at night

Table 1 lists the genes for ubiquitin ligases that were reported as significantly elevated at least 2-fold in rat pineals sampled at night versus those sampled during the day. Rhobtb3 (Rho related BTB domain containing 3), is a ubiquitin ligase associated with the Golgi apparatus [60]. Although Rhobtb3 is a member of the Rho GTPase family, it reportedly functions as an ATPase [60]. One of its known substrates is cyclin E; as such it contributes to regulation of transition of the cell cycle from the S phase to the G2 phase [61]. Inhibition of Rhobtb3 results in accumulation of cells in the S phase [61]. The Hartley dataset show that this ligase was increased 3.87-fold at night, at a very high level of significance (FDR < 4.64^E-42^), and that this rise was prevented by surgical removal, or disconnection, of the superior cervical ganglia. It was also stimulated in vitro by DBcAMP (3.08-fold, FDR < 1.75^E-63^) and by NE (4.13-fold, FDR < 1.25^E-149^) at very high levels of statistical significance (Hartley supplemental dataset 3). These findings indicate that Rhobtb3 could play an important role in light/dark-induced adrenergic modulation of pineal function. More information on additional substrates of this ubiquitin ligase will probably be required before its role in the pineal gland is understood.

Ubr4 (ubiquitin protein ligase E3 component N-recognin 4) is an ubiquitin ligase that binds to calmodulin but was originally named for its association with the retinoblastoma protein (RB); it was first named p600 retinoblastoma protein-associated factor. Although RB is known for its association with retinoblastoma when its gene is doubly mutated, loss of RB is also associated with other cancers [62] including these in lung, bone, breast and urinary bladder. Ubr4 also binds to Itpr1 (inositol trisphosphate receptor isoform 1), which regulates calcium release from the endoplasmic reticulum [63]. Furthermore, it binds to papillomavirus E7 protein from a number of different HPV (human papilloma virus) subtypes [64]. The Ubr4 ligase was increased at night (2-fold) in the Hartley dataset (Table 1). The nighttime increase was prevented by surgical removal of the superior cervical ganglia. Of the ubiquitin ligases increased more than 2-fold at night the rise in Ubr4 was most significant (FDR = 2.07^E-152^). It is not clear that an E3 ligase which regulates the stability of the RB tumor suppressor should be physiological important in the pineal gland. The fact that retinoblastoma is sometimes associated with a pineal tumor (trilateral retinoblastoma) suggests a developmental or evolutionary relationship [65]. An animal model for trilateral retinoblastoma has been reported in which pineal tumors accompany retinoblastoma [66].

FBXO33 (F-Box protein 33) is encoded by an apoptosis-inducible gene [67]. One substrate that has been identified for the FBXO33 ligase is the YB-1 (Y-box binding protein 1) protein which it ubiquitinates in preparation for degradation by the proteasome. YB-1 plays a significant role in regulating cell cycle proteins, cyclin A and B1 [68], and functions as a regulator of mRNA stability [69]. It is a DNA binding protein and contributes to cell differentiation [70]. Lutz et al. [67] describe it as a multifunctional regulator.

A number of members of the KLHL family of genes was shown to be significantly increased at night in the Hartley dataset. The proteins coded by these genes include at least 42 members in humans [71]. *Klhl30* (kelch like family member 30) is an ubiquitin ligase that is elevated at night (3.81-fold, p < 6.94^E-10^) in the Hartley dataset, but only in rats with intact superior cervical ganglia. It was also significantly stimulated by NE (1.88-fold, p < 0.014). Although the function of this ubiquitin ligase is not understood, it has been associated with circadian clock genes [72].

Three additional members of the KLHL family are found in Table 1. *Klhl4*, *Klhl14*, and *Klhl29* were all significantly higher at night by at least 2-fold. Furthermore *Klhl21* was significantly elevated at night by 1.98-fold. The cellular functions of KLHL4, KLHL14 and KLHL29 are not known whereas that of KLHL21 has been investigated. One substrate for KLHL21 is Aurora B. KLHL21 ubiquitinates Aurora B prior to anaphase and is necessary for cytokinesis [73]. It is not clear why this mechanism of regulation of mitosis would be important in the pineal gland given that pinealocytes exhibit a low replicative activity.

HERC4 (HECT and RLD domain containing E3 ubiquitin protein ligase 4) has been described as a guanine nucleotide exchange factor for G proteins [74]. It physiological function, however, is not established. *Herc4* expression was elevated 2.76-fold in the Hartley dataset.

The expression of three members of the KLHL family was found to be significantly depressed by at least 2-fold: these include *Klhl10*, *Klhl35* and *Klhl36* (Table 2). In addition the expression of two members of this family were significantly reduced by 1.5 to 2-fold, i.e., *Klhl12* and *Klhl19* (also known as *KEAP1*). Klhl10 is part of an E3 ubiquitin ligase that activates caspase in spermatids and therefore contributes to spermatid remodeling. This interaction occurs on the surface of mitochondria and is linked to the Krebs cycle [75]. Little is known of KLHL35 or KLHL36, although the gene for KLHL35 has been associated with renal and hepatocellular carcinoma [76, 77]. KLHL12 reportedly ubiquitinates the dopamine D4 receptor [78, 79]. KEAP1 is an ubiquitin ligase that modulates the cellular response to oxidative stress [71, 80, 81]. As such KEAP1 is the best-known ubiquitin ligase of the KLHL family. It has at least three known substrates, NRF2 (nuclear factor, erythroid 2 like), IKKΒ (inhibitor of nuclear factor kappa-B kinase subunit Beta) and BCL-2 (B-cell lymphoma apoptosis regulator 2) [82].

Other E3 ligases whose expression was reduced at nighttime at least two-fold in the Hartley dataset include *Trim9*, *Dtx4*, *Znf238*, *Abtb2*, *Trim45*, *Rnf19b*, *Rnf128*, *Rnf182*, *Peli2*, *Fbox23*, *Traip*, and *March4*. These ubiquitin ligases regulate the stability of a variety of substrates via pathways that are only partially understood in tissues other than the pineal gland. TRIM9 has been reported to regulate development of the hippocampus [83]. TRIM45 regulates a protein in the PKC pathway [84] and modulates NF-κB-induced transcription [85]. ABTB2 (Ankyrin repeat and BTB domain contain 2) has been described as a tumor suppressor and reportedly inhibits alpha-synuclein in the substantia nigra [86]. The function of some are unknown, e.g. FBXO23. ZNF238 is a transcriptional repressor [87] during neuronal development. Several of the other E3 genes whose expression were seen to be reduced at night play a role in immune response signaling (*Dtx4*, *Trim45*, *Rnf19B*, *Rnf128*, *Rnf182*, *Peli2*).

### Deubiquitinases as adrenergic targets in the pineal gland

The expression of genes coding for five deubiquitinases (DUBs) were significantly elevated (greater than 2-fold) in the pineal gland at night: these include *Usp2*, *Usp16*, *Usp 26*, *Usp29* and *Usp38*. These day/night differences were not observed in rats subjected to surgical removal of the superior cervical ganglia. Expression of three of these genes (*Usp2*, *Usp16* and *Usp38*) were also significantly augmented by DBcAMP and/or NE. Expression of *Otud7b*, a DUB of the ovarian tumor class, was significantly depressed at night. We discuss functional aspects of these DUBs below.

Among the proteins that USP2 (ubiquitin specific protease 2) deubiquitinates is the circadian clock protein PER1. In doing so it regulates the entry of PER1 into the nucleus [88]. Since *Usp2* expression was up-regulated at night in the Hartley dataset, based on the findings of Yang et al. [88] we would expect an increased nuclear accumulation of Usp2 at night in the rat pineal gland. A role for Usp2 in regulation of circadian rhythms and Per1 was previously reported by this group based on observations on tissues of *Usp2* knockout mice [89]. USP2 is also required for TNFalpha induced NF-kB signaling in kidney cells [90]. USP16 acts as a DUB for histone H2A [91]. It is required for normal cell division [92]. It reportedly interacts with the ubiquitin ligase HERC2 (Hect domain E3 ligase 2) to modulate the cellular response to DNA damage [93]. It is also a regulator of embryonic stem cell differentiation [94]. USP29 is also required for normal cell division. It regulates the stability of one of the DNA damage checkpoint proteins, claspin [95]. Little functional information is available concerning USP38. OTUD7B may be involved in regulation of TRAF3 and NF-κB and hence the immune response [96].

Of the DUBs stimulated by DBcAMP and NE in the Hartley dataset the expression of *Yod1* (aka ovarian tumor protease, aka HIV-1 induced protease 7) was increased (5-fold by DBcAMP) more than any other. The expression of *Yod1* was also significantly elevated during the night (although not at the 2-fold level). Functionally YOD1 is part of a mechanism for translocating misfolded proteins from the endoplasmic reticulum back to the cytoplasm in preparation for degradation by proteasomes [97, 98]. Two DUBs, Senp7 (sentrin specific peptidase 7) and Parp11 (poly ADP-ribose polymerase 11) were significantly reduced by DBcAMP. While a number of proteins are up-regulated at night in the pineal gland, it has not been established that this organ secretes a protein of major functional significance. The number of E3 ligases and DUBs significantly modified by adrenergic stimulation, however, suggests that adrenergic regulation of transcription and regulation of degradation of specific proteins in the pineal may be more important than previously available data indicate.

### Adrenergic signal transduction (cAMP/PKA signaling) in the pineal gland and the ubiquitin proteasome system

Adrenergic activation of cAMP/PKA signaling inhibits proteasome-dependent proteolysis in muscle [99]. Huang et al. [100] reviewed the literature relating cAMP/PKA signaling to the UPS. They document and provide evidence showing that cAMP/PKA signaling regulates the UPS by phosphorylating ubiquitin ligases, proteins comprising proteasome subunits and other proteins required in the UPS pathway. The ubiquitin ligases regulated by PKA included CHIP (carboxy terminus of HSP70-interacting protein), MuRF-1 (muscle specific ring finger protein 2), and ATROGIN-1 (muscle atrophy F-box protein) (aka FBXO32). The Hartley dataset provides evidence that adrenergic activation of the pineal gland stimulates the activity of many ubiquitin ligases. Of the 34 ubiquitin ligases whose expression was increased at night by 2-fold or greater (Table 1), 28 were significantly stimulated by DBcAMP in culture. In addition, a number of ubiquitin ligases were augmented by DBcAMP but not significantly influenced by the light/dark cycle. All ubiquitin ligases stimulated by DBcAMP (greater than 2-fold) are shown in Table 4 and those inhibited (more than 2-fold) by DBcAMP in Table 5. The level of the significant change in response to DBcAMP varied from p < .05 (*Klhl30*) to p < 2.93E^-273^ (*Herc4*). The *Herc4* ligase was also promoted by NE stimulation in vitro (p < .3.12 E^-68^). Clearly, the pineal provides a unique model to study adrenergic innervation and the expression of genes for ubiquitin ligases.

PKA phosphorylates subunits of the alpha and beta rings of the 20S proteasome in heart and liver [101]. Furthermore, PKA stimulates the peptidase activities of the three catalytic enzymes of the proteasome, B_1_ caspase-like activity, B_2_ trypsin-like activity, and B_5_ chymotrypsin-like activity [101] in these organs.

### Melatonin and the ubiquitin proteasome system

There are numerous reports documenting that melatonin inhibits cAMP under various experimental conditions [102–104]. Based on the model of Huang et al. [100], a reasonable prediction is that under experimental conditions where melatonin inhibits cAMP/PKA, it would inhibit the activity of proteins phosphorylated by PKA including a number (probably a large number) of ubiquitin ligases.

Melatonin could also influence the activity of a large number of ubiquitin ligases if it acted as a neddylation inhibitor. While there is no direct evidence for this, it should be noted that the indole ring of melatonin is similar to a major component of an inhibitor reported for the NEDD8-activating enzyme [105]. The role of neddylation and deneddylation in regulation of the ubiquitin ligases has been reviewed and illustrated by Pierce et al. [106]. A large number of ubiquitin ligases are deactivated through deneddylation by the COP9 signalosome (CSN) complex.

Similarities in the action of melatonin and proteasome inhibitors in various tissues have been noted [107]. The antioxidant action(s) of melatonin have been related to the activity of the E3 ubiquitin ligase Keap1 [80]. Melatonin also interacts with the HIF-1 (hypoxia-inducible factor 1) oxygen-sensing mechanism in peripheral tissues. The von Hippel-Lindau ubiquitin ligase plays a key role in this mechanism [108]. There are no studies in which melatonin has been tested as to its effects on the activity or tissue levels of ubiquitin ligases. It would be interesting to see data on whether melatonin inhibits the activity of ubiquitin ligases in the pineal gland and/or in peripheral tissues.

### Functional significance

The Hartley dataset provides some remarkable findings concerning the UPS in the pineal gland. The data show that the expression of genes for six ubiquitin conjugases and more than 50 ubiquitin ligases exhibited a greater than 2-fold variation (increase or decrease) in night versus day expression. Most of these changes were prevented by removal of the superior cervical ganglia and could be mimicked in organ culture by the addition of DBcAMP or NE. Thus the Hartley report establishes a functional relationship between the adrenergic innervation of the pineal gland and transcription of numerous genes coding for ubiquitin ligases or deubiquitinases. Furthermore the Hartley data provide evidence for a 35-fold increase in the expression of a gene that codes for SIK1, a protein that does double duty as a kinase and as a protein that interacts with ubiquitinated proteins destined for proteasomal degradation. Further studies are needed to determine the role of SIK1 in autonomic-induced day-night differences in the proteins of the ubiquitin proteasome pathway.

The ubiquitin ligases and deubiquitinases modulated by the beta-adrenergic innervation of pineal cells provide a cellular on/off switch for rapidly changing cellular levels of melatonin and similarly for modifying pineal concentrations of the thyroid hormone, T3, by regulating levels of Dio2. It has yet to be determined whether pineal Dio2 influences circulating levels of T3. Another fundamental question arising from the present study is whether the day/night differences in Dio2 are independent of pineal concentrations of melatonin. It has not been determined whether melatonin modulates the activity of E3 ligases which regulate the stability of Dio2 within the pineal gland, or, for that matter, whether melatonin modulates the activity of any other E3 ligases that are subject to day/night variations.

The large number of pineal ubiquitin ligases whose expression is influenced by the light/dark cycle suggests that the pineal gland contains several additional proteins (other than those regulating melatonin and T3 levels) whose stability is regulated by ubiquitin ligase/deubiquitinase on/off molecular switches. If the number of E3 ligases influenced by the adrenergic system of the pineal gland is any indication, one or more major molecular functions of the pineal is yet to be identified. One potentially important function based on the Hartley dataset would be that of a regulator of potassium ion channels. Some of the variations in E3 ligases could relate to local housekeeping functions of the cell, while others may regulate substrates that enter the venous system and act peripherally or enter the CSF of the ventricular system [109] to act on the central nervous system.
